# Theoretical Study of Radical Inactivation, LOX Inhibition, and Iron Chelation: The Role of Ferulic Acid in Skin Protection against UVA Induced Oxidative Stress

**DOI:** 10.3390/antiox10081303

**Published:** 2021-08-18

**Authors:** Ana Amić, Jasmina M. Dimitrić Marković, Zoran Marković, Dejan Milenković, Žiko Milanović, Marko Antonijević, Denisa Mastiľák Cagardová, Jaime Rodríguez-Guerra Pedregal

**Affiliations:** 1 Department of Chemistry, Josip Juraj Strossmayer University of Osijek, Ulica cara Hadrijana 8A, 31000 Osijek, Croatia; 2 Faculty of Physical Chemistry, University of Belgrade, Studentski trg 12–16, 11000 Belgrade, Serbia; markovich@ffh.bg.ac.rs; 3 Department of Science, Institute for Information Technologies, University of Kragujevac, Jovana Cvijića bb, 34000 Kragujevac, Serbia; zmarkovic@uni.kg.ac.rs (Z.M.); deki82@kg.ac.rs (D.M.); ziko.milanovic@uni.kg.ac.rs (Ž.M.); mantonijevic@uni.kg.ac.rs (M.A.); 4 Department of Chemistry, Faculty of Science, University of Kragujevac, Radoja Domanovića 12, 34000 Kragujevac, Serbia; 5 Institute of Physical Chemistry and Chemical Physics, Department of Chemical Physics, Slovak University of Technology in Bratislava, Radlinského 9, SK-812 37 Bratislava, Slovakia; denisa.cagardova@stuba.sk; 6 In Silico Toxicology, Institute of Physiology, Charité—Universitätsmedizin Berlin, Charitéplatz 1, 10117 Berlin, Germany; jaime.rodriguez@charite.de

**Keywords:** ferulic acid, 5-hydroxyferulic acid, density functional theory (DFT) and molecular docking, radical scavenging, PCET, radical-radical coupling, minimum energy crossing point (MECP), water assisted tautomerization, lipoxygenase, iron chelation

## Abstract

Ferulic acid (FA) is used in skin formulations for protection against the damaging actions of the reactive oxygen species (ROS) produced by UVA radiation. Possible underlying protective mechanisms are not fully elucidated. By considering the kinetics of proton-coupled electron transfer (PCET) and radical-radical coupling (RRC) mechanisms, it appears that direct scavenging could be operative, providing that a high local concentration of FA is present at the place of ^•^OH generation. The resulting FA phenoxyl radical, after the scavenging of a second ^•^OH and keto-enol tautomerization of the intermediate, produces 5-hydroxyferulic acid (5OHFA). Inhibition of the lipoxygenase (LOX) enzyme, one of the enzymes that catalyse free radical production, by FA and 5OHFA were analysed. Results of molecular docking calculations indicate favourable binding interactions of FA and 5OHFA with the LOX active site. The exergonicity of chelation reactions of the catalytic Fe^2+^ ion with FA and 5OHFA indicate the potency of these chelators to prevent the formation of ^•^OH radicals via Fenton-like reactions. The inhibition of the prooxidant LOX enzyme could be more relevant mechanism of skin protection against UVA induced oxidative stress than iron chelation and assumed direct scavenging of ROS.

## 1. Introduction

As a protective barrier of the body, human skin is continuously exposed to solar UV radiation. Prolonged exposure may result in oxidative stress caused by radiation induced generation of reactive oxygen species (ROS, e.g., ^1^O_2_, ^•^OH, O_2_^•−^, O_3_, and H_2_O_2_). The main adverse health effects of UV radiation are premature skin aging, immune suppression, increased risks of skin cancer, and eye disorders and diseases [1,2].

The hydroxyl radical (^•^OH) can be produced by solar ionizing radiation and UV radiations. High energy γ-rays and X-rays produce ^•^OH by homolytic fission of water. Lower energy UVB is absorbed in the epidermis and dermis of the skin and can generate ^•^OH by homolytic fission of H_2_O_2_,widespread in cells [3]. UVA penetrates deeper into the dermal layer and the circulating blood cells [3,4,5] and enables oxygen molecules to produce ^•^OH via radiation induced ROS generating pathways such as: O_2_ → O_2_^•−^ → H_2_O_2_ → ^•^OH [1,6,7].

Among ROS, ^•^OH is the most harmful free radical to living organisms as it can be formed intracellularly via iron-catalysed Fenton reaction [8]. As the main source of biological damage in the cell, it reacts with any molecule at the site of its formation, mainly with the diffusion-limited rate constant (in the order of 10^7^–10^10^ M^−1^ s^−1^). Urea is a rare exception: its rate constant is low for a reaction with ^•^OH and amounts 7 × 10^5^ M^−1^ s^−1^ [7]. Damage of cell proteins, lipids, and DNA contributes to the development of a variety of chronic and degenerative diseases [8]. Conversely, ^•^OH may also participate in many important biological reactions [9]. For example, it is produced in low (moderate) levels by phagocytes for bactericidal and cytotoxic activities.

Several enzymes contribute to the production of ROS. Human skin represents a tissue with abundant and diverse lipoxygenase (LOX) activities. LOX, a class of non-heme iron containing enzymes [10,11], contributes to the generation of various ROS including fatty acid hydroperoxide, peroxyl, alkoxyl, and O_2_^•−^ radicals [10,12,13]. Two O_2_^•−^ species can react via dismutation reaction to generate H_2_O_2_: O_2_^•−^ + O_2_^•−^ + 2 H^+^ → H_2_O_2_ + O_2_ [7]. In an iron-catalyzed Haber-Weiss reaction, O_2_^•−^ and H_2_O_2_ produce ^•^OH [14]. LOX isozymes may be involved in the modulation of epithelial proliferation, wound healing, inflammatory skin diseases, and cancer [11].

The skin is equipped with cell protective enzymatic antioxidants such as superoxide dismutase, catalase, glutathione peroxidase, and nonenzymatic antioxidants such as vitamin C and E, ingested via diet [15]. Oxidative stress appears when skin’s endogenous antioxidant defence ability is overwhelmed by the UV mediated generation of ROS. To suppress such a scenario, topical antioxidant lotions may be beneficial. Antioxidants may prevent ROS induced skin damage only when they are present in relevant concentration in situ (at the skin’s surface but also inside the skin) before Sun exposure [16,17]. Ferulic acid (FA) is well absorbed by the skin and is widely used in skin formulations [2,17,18,19,20,21,22,23,24,25,26]. FA is a widespread dietary phytochemical that can be found in cereal bran, citrus juice, coffee, and numerous vegetables and fruits [18,27,28]. A contribution to skin protection could also be expected from orally taken FA [24], as well as from FA produced by gut microbiota in large intestine from tea catechins, esterified hydroxycinnamates, polymeric proanthocyanidins, and acylated flavonoid glycosides [29,30]. FA is an abundant and highly bioavailable colon catabolite of these compounds [31,32].

Underlying mechanisms by which FA may suppress UV-induced ^•^OH radical damage to skin cells are not well understood. In this report, the possible fate of FA under ^•^OH radicals attack was kinetically investigated. The success of any molecule to inactivate ^•^OH depends on its concentration at sites of ^•^OH generation, in relation to that of all other nearby competing biomolecules [33]. We assumed that by maintaining a high local concentration within the skin after topical application and/or a sufficient concentration in the systemic circulation [25,34,35], FA could be able to intercept more than a minor percentage of generated ^•^OH. Inactivation of ^•^OH radicals was investigated via several mechanisms including hydrogen atom transfer (HAT), proton coupled electron transfer (PCET), radical adduct formation (RAF), and radical-radical coupling (RRC) [34,36,37].

In principle, the only efficient protection mechanism against highly reactive free radicals such as ^•^OH is to prevent their formation [38,39,40]. It should be noted that there are no direct enzymatic mechanisms for specific ^•^OH scavenging [9]; so, inhibition of prooxidant enzymes that produce the ROS involved in non-enzymatic generation of ^•^OH could be beneficial. Inhibition of the LOX enzyme, one of several enzymes that catalyze ROS formation, was considered by molecular docking analysis. The molecular docking technique is able to predict the binding modes of FA and its studied derivative, as well as the effect of the hydroxyl group(s) of ligands on protein-binding properties. 

Another antioxidant mechanism is based on the ability of natural (poly)phenolics to chelate catalytic transition metals ions, giving rise to stable complexes that, entrapping catalysts, prevent free radical generation [41]. The potency of FA and its studied derivative to chelate Fe^2+^ ion involved in ^•^OH formation via Fenton and Haber-Weiss reactions was investigated. Chelator species responsible for Fe^2+^ ion sequestration, preferred coordination sites, and structural features of formed chelates were identified. 

The main goal of this study is the examination of the possible fate of ferulic acid under ^•^OH radicals attack. Underlying scavenging mechanisms are analyzed for both FA and its reaction product 5-hydroxyferulic acid (5OHFA). To achieve this, two-state reactivity that involves spin inversion in the RRC rate-determining step was considered. Also, the inhibitory activity of those compounds against the LOX enzyme was tested as well as their potency in catalytic Fe^2+^ ion binding.

## 2. Materials and Methods

### 2.1. DFT Calculations

The DFT method with M06-2X functional and 6-311++G(d,p) basis set implemented in the Gaussian 09 program package [42] was used for geometry optimisations and frequency calculations. The M06-2X functional [43] provides very good performance for thermochemistry and barrier heights [44] and was proven as very suitable for study of thermodynamics and kinetics of free radical inactivation by polyphenols [36]. After finding the transition state (TS) structure, which possesses a single imaginary frequency, an intrinsic reaction coordinate (IRC) calculation was carried out on both sides of the TS to find two related energy minima: the reactant complex (RC) and the product complex (PC). Both RC and PC structures were further optimized to obtain geometries with no imaginary frequency. All calculations related to radical scavenging mechanisms were performed in the gas-phase at 298.15 K.

The conventional transition state theory (TST) was applied to calculate rate constants (*k*^TST^) for studied reactions at the M06-2X/6-311++G(d,p) level using the Eyringpy program [45]:

(1) $$k^{\mathrm{TST}}=\sigma \kappa \frac{k_{B}T}{h}e^{-\left(G^{\neq }\right)/RT}$$

where σ, κ, *k*_B_, *T*, *h*, Δ*G*^≠^, and *R* represent the number of equivalent reaction pathways (i.e., reaction path degeneracy), Eckart tunneling corrections [46], Boltzmann constant, temperature, Planck constant, Gibbs free energy of activation, and gas constant, respectively. An estimate of the Eckart tunneling correction considers RC and PC [47].

To differentiate the HAT from the PCET mechanism, several methods have been proposed [48,49]. We used the simplest one, which estimates the character of TS’s singly occupied molecular orbital (SOMO). In addition, by using the AIMAll program package [50], the QTAIM charge of transferring hydrogen in the TS structure was considered.

The minimal energy crossing point (MECP) is initially predicted as described by Kaur and co-workers [51], and then calculated using the EasyMECP program [52].

The complexation reactions of FA with the hydrated Fe^2+^ ion were evaluated using M06 functional because it is more appropriate for transition metal chemistry [53]. The 6-311++G(d,p) basis set was used. The SMD solvent continuum model [54] was used to represent the aqueous environment. 

### 2.2. Molecular Docking Simulation

The AMBER force field implemented in the AutoDock 4.0 software package [55] was used to predict the binding mode between the LOX enzyme and investigated compounds, FA and 5OHFA. The crystal structure of the LOX enzyme complex with 13(*S*)-hydroperoxy-9(*Z*),11(*E*)-octadecadienoic acid (13-HPOD) was taken from the Protein Data Bank (RSCB) with the PDB code 1IK3 [56]. Discovery Studio 4.0 [57] was used to prepare protein structures in a form advisable for molecular docking simulation. In addition, this software was applied for analysis of the obtained results after the molecular docking simulation. Polar hydrogen atoms were added using the hydrogen module in the AutoDockTools (ADT) graphical interface. The Kollman united atom partial charges were used for defining partial atomic charges. The bonds in investigated compounds were set to be rotatable with 5 (FA) and 6 (5OHFA) active torsions. Grid maps were determined using the AutoGrid module with a grid box of dimension 60 × 60 × 60, with the point separated by 0.375 Å (grid-point spacing). Grid centre coordinates correspond to the defined active location and amount to 23.88 × 1.75 × 13.76. Finally, the Lamarckian Genetic Algorithm (LGA) was implemented for the rigid-flexible docking simulation. For this purpose, the 10 conformers of ligand were considered.

## 3. Results and Discussion

### 3.1. Inactivating the ^•^OH Radical by FA via the PCET Mechanism

**Figure 1:**
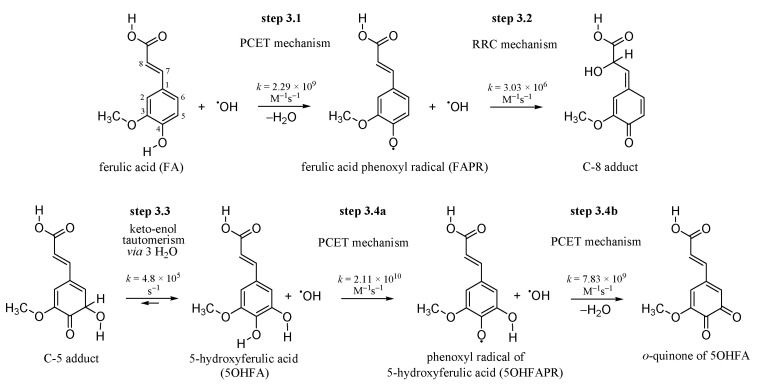
Presumed steps of fate of ferulic acid under ^•^OH radicals attacks. Kinetic data are obtained using the M06-2X/6-311++G(d,p) level of theory in the gas-phase.

According to the facts mentioned in Section 1, it could be predicted that the ^•^OH radical may strip the H-atom from the phenolic OH group at a rate approaching, or in the range of, diffusion-limited reactions. Figure 1 summarizes the proposed fate of FA under ^•^OH radicals attacks. The reaction between FA and ^•^OH (step 3.1 in Figure 1) proceeds by first making RC, followed by TS and PC, and finally leads to the separated products (i.e., ferulic acid phenoxyl radical (FAPR) and water). The optimized geometries of stationary points obtained from the M06-2X/6-311++G(d,p) calculations are presented in Figure S1.

The results of the kinetic analysis performed are presented in Table S1a. The calculated rate constant *k*^TST/Eck^ = 2.29 × 10^9^ M^−1^ s^−1^ indicates that the reaction is diffusion-limited.

**Figure 2:**
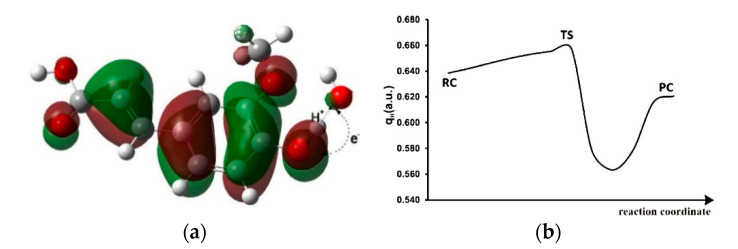
(**a**) TS structure of the reaction of the 4-OH group of FA with the ^•^OH radical accompanied by the corresponding SOMO. (**b**) The change of charge on transferring the H-atom along the reaction coordinate for the reaction between FA and ^•^OH radical.

The simplest method to ascertain the mechanism by which the ^•^OH radical abstracts H-atom from the 4-OH group of FA is based on the nature of the SOMO at the TS structure [49]. An H-atom transfer that involves two heteroatoms should be PCET rather than HAT [58]; closer inspection of Figure 2 confirms this fact. As demonstrated in Figure 2a, the SOMO in TS is not localized along the O-donor∙∙∙H∙∙∙O-acceptor axis and the orbitals on the hydrogen donor and acceptor atoms are nearly orthogonal to the transition vector, revealing that the H-atom transfer occurs mostly via a PCET pathway. The proton is transferred between lone pairs of electrons in σ orbitals on the oxygen, while the electron transfers from the 2*p* lone pair of the oxygen of FA to the SOMO of the ^•^OH radical.

The change of QTAIM positive charge along the IRC of the studied reaction path is shown in Figure 2b. The migrating hydrogen atom shows a substantial positive charge, typical for proton migration [59,60]. Positive charge in the RC (qH = 0.64 a.u.) increases in TS (qH = 0.66 a.u.) and then decreases in the PC (qH = 0.62 a.u.). This confirms that hydrogen abstraction from the 4-OH group of FA by the ^•^OH radical takes place via the PCET mechanism. The HAT proceeds with a small positive charge (qH < 0.5 a.u.) for the transferring H-atom in the TS structure [61]. 

### 3.2. Inactivation of the ^•^OH Radical by FAPR via the RRC Mechanism

**Figure 3:**
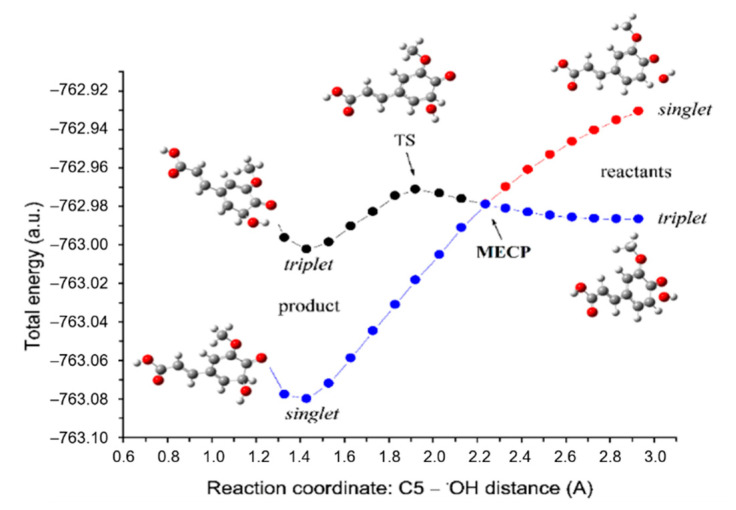
Energy profiles for RRC pathways of the C5 site of FAPR with the ^•^OH radical. Red and black lines indicate singlet and triplet states, respectively. The proposed reaction path is denoted by the blue line.

Regardless of the operative H-atom abstraction mechanism by which FA inactivates the ^•^OH radical, the final products are the same (i.e., FAPR and water). The mechanism by which FAPR may inactivate the ^•^OH radical is RRC (step 3.2 in Figure 1). In our recent article [37], we found that the RRC reaction between the C1, C3, C5, and C8 sites of FAPR with a set of free radicals is highly thermodynamically favourable; here, we are dealing with kinetics of this reaction mechanism. The procedure for estimating the rate constant related to the RRC mechanism is exemplified below in the case of the reaction between the C5 site of FAPR with the ^•^OH radical (Figure 3).

As separated reactants, FAPR and the ^•^OH radical exist as doublets. Reaction between them occurs on the two potential energy surfaces (PES), a reactivity phenomenon named the two-state reactivity [62] (Figure 3). Such a reaction pathway involves a change in the spin state and is formally forbidden [63]. As reaction proceeds, the singlet state energy constantly decreases. In triplet state, reactants are more stable than in singlet state, and approaching each other triplet state energy increases achieving its maximum at triplet TS. However, reaction does not take place via this energy more demanding TS because a lower energy reaction path exists via intersection of the two PESs, called the minimal energy crossing point (MECP). At the MECP, the spin inversion occurs (triplet → singlet) enabling production of a much more stable product in singlet state. 

The geometry of the MECP (i.e., the conformation of the molecular system at the intersection between the reactant (triplet) and the product (singlet) PES), plays the same role as the transition state in reactions proceeding on a single PES [64]. Locating the MECP structure is necessary for calculating the reaction rate constant [65,66]. To identify the MECP, firstly, the reaction pathway is traced toward the RC and the PC starting from the triplet TS, and each point encountered along the IRC path of the TS was submitted to a single point energy calculation with the singlet electronic state [51]. The C5−^•^OH distance was selected as the reaction coordinate. By this procedure, the MECP is predicted to appear at the C5−^•^OH distance of 2.09917 Å (Figure S2). By another approach [67] (i.e., full scan procedure starting from RC geometry in singlet state and going to PC (using the C5−^•^OH distance as the reaction coordinate)), obtained MECP appears at a distance of 2.23961 Å (Figure 3). Afterwards, to achieve a more precise estimate of the rate constant, the structure closest to the initially estimated MECP was used as a starting geometry in the MECP search using Harvey’s original code [64], implemented in the EasyMECP program [52]. In this way, the more accurate MECP appears at the C5−^•^OH distance of 2.23494 Å and is used as the TS in estimating the rate constant. The obtained results are summarized in Table 1.

As found in Table 1, the favoured path for RRC reaction is the C-8 site of FAPR. The overall rate constant $k_{\mathrm{overall}}^{\mathrm{TST}/\mathrm{Eck}}$ (i.e., the sum of the rate constants related to considered reaction paths) amounts to 3.03 × 10^6^ M^−1^ s^−1^. This result in not close to diffusion driven reactions, which should be expected for RRC reactions [68]. It should be noted that a computational error in barrier height of 1 kcal/mol produces nearly one order of magnitude difference in the calculated rate constant [69]. More importantly, the same is true for MECP determination, which is not a stationary point. Low imaginary frequency of MECP (which here play the role of TS) indicates a lower *k* value. Those facts could be the main reason for the underestimated rate constants listed in Table 1. 

Addition of the ^•^OH radical to the C5 site of FAPR (Figure 1) results in a product which may be considered as a keto form of 5-hydroxyferulic acid (5OHFA). The calculated Δ_r_*G* value of −28.2 kcal/mol indicates keto-enol tautomerism, which results in 5OHFA, as thermodynamically highly feasible. In the forthcoming section, the kinetics of this process is discussed.

### 3.3. Keto-enol Tautomerization: Proton Transfers along Hydrogen Bonds 

Amongst RRC products, the adduct of the ^•^OH radical with C-5 site of FAPR deserves particular attention because, via keto-enol tautomerization, it gives the catecholic compound 5OHFA (step 3.3 in Figure 1). The catechol group (two vicinal OH groups) is a well-known supreme free radical scavenging moiety [70].

Results of performed kinetic analysis of the tautomerization reaction are provided in Table 2. The calculated value of Δ*G*^≠^ is too high; consequently, the *k*^TST/Eck^ is too low for proton rearrangement in the process of tautomerization. It is known that Δ*G*^≠^ decreases with increasing numbers of catalytic water molecules during the keto-enol tautomerization process [71,72]. Thus, in the process of tautomerization of acetylacetone assisted by three water molecules, small values for Δ*G*^≠^ were obtained [71]. In this article, 1–3 water molecules are introduced, which are appropriately linked to the substrate molecule. As demonstrated in Table 2 and Figure S3, keto-enol tautomerization via one water molecule produces a huge diminution of the Δ*G*^≠^. The water molecule forms a bridge for the intramolecular proton transfer from the α-carbon to the oxo group. The hydrogen bond network of the water molecules highly stabilizes TS, leading to the decrease of Δ*G*^≠^. Proton transfer via the hydrogen-bond lattice produces enol form. If it occurs via H-bonds connected by three water molecules, the Δ*G*^≠^ decreases significantly and consequently the rate constant *k*^TST/Eck^ increases (Table 2, Figures S3 and S4). Optimized geometries of keto form, TS, and enol form with the three involved water molecules are presented in Figure S5. 

The data presented in Table 1 indicate that the product of the RRC reaction mechanism at C5 site (keto form of 5OHFA) is not the preferred one. However, sequential keto-enol tautomerization proceeds fast (*k*^TST/Eck^ = 4.8 × 10^5^ s^−1^, Table 2), shifting the equilibrium to production of 5OHFA.

5OHFA is a natural product and was found in *Zea mays* L. and *Hordeum vulgare* L. as a cell wall bound ester [73]. It is well known that this compound is effective in the scavenging of DPPH^•^ free radicals in the in vitro assay [74]. To the best of our knowledge, the kinetics of its free radical scavenging potency have not been investigated.

### 3.4. 5OHFA Inactivates Two ^•^OH Radicals via Double PCET Mechanism

If the abovementioned scenario appears on the skin, formed 5OHFA will suffer from ^•^OH radical attacks too. The catecholic moiety of 5OHFA enables the scavenging of two ^•^OH radicals (step 3.4a and step 3.4b in Figure 1). The first one is scavenged by the 4-OH group of 5OHFA. The optimized geometries of stationary points (i.e., RC, TS, and PC, for the H-atom abstraction via PCET mechanism) are presented in Figure S6. From the obtained kinetic data listed in Table S1b, it is obvious that this is a diffusion-controlled reaction (*k*^TST/Eck^ = 2.11 × 10^10^ M^−1^ s^−1^).

**Figure 4:**
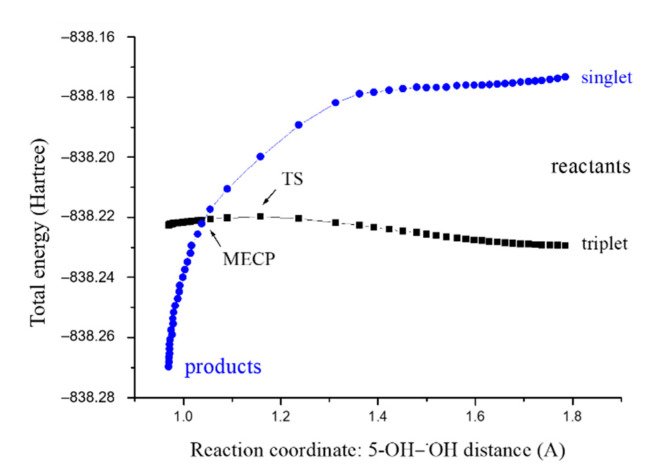
Energy profiles for the PCET pathways of the 5-OH site of phenoxyl radical of 5-hydroxyferulic acid with ^•^OH radicals in the singlet (blue line) and triplet (black line) states.

The reaction product phenoxyl radical of 5-hydroxyferulic acid (5OHFAPR) may scavenge another ^•^OH radical. The reaction between those two radicals occurs on the two PESs (Figure 4). The reactant complex in triplet state is much more stable than RC in singlet state. As reactants approach each other, reaction proceeds through the energy barrier, (i.e., TS in triplet state). The spin inversion (MECP) occurs after passage of the TS; therefore, MECP does not affect the rate constant, contrary to the situation depicted in Figure 3. As demonstrated in Figure 4, after the MECP, reaction proceeds toward much more stable products in the singlet state. 

Figure 4 was created by the procedure described in Section 3.2 [51]. Such predicted MECP appears at the 5-OH−^•^OH distance of 1.04248 Å. The more precise MECP calculated using the EasyMECP program [52] amounts to 1.02975 Å. Regardless of those values, MECP has no impact on the reaction kinetics because it is located at a lower energy on the product side of the respective reaction barrier (triplet TS). Optimized geometries of involved stationary points are presented in Figure S6, and the results of kinetic analysis in Table S1c. As demonstrated in Table S1c, the second H-atom abstraction is also a diffusion-controlled process (*k*^TST/Eck^ = 7.83 × 10^9^ M^−1^ s^−1^). 

Obtained results of performed kinetic analysis indicate that FA and 5OHFA, as well as their corresponding phenoxyl radicals under ^•^OH radicals attack, may inactivate ^•^OH radicals by diffusion-controlled reaction rates. If such scenario occurs in situ (i.e., at the site of ^•^OH radicals formation where concentration of FA is high), investigated pathways may contribute to skin protection from UVA radiation. 

### 3.5. Molecular Docking

As emphasized in the Section 1, it appears that the inhibition of prooxidant enzymes might block the UVA-induced skin damage caused by ^•^OH radicals. To understand the binding efficiency of the investigated compounds FA and 5OHFA, to the LOX enzyme, it is necessary to discuss the thermodynamic parameters obtained after the molecular docking simulation. The results of the important thermodynamic parameters are provided in Table 3. The negative values of free binding energy (Δ*G*_bind_) and small values of the constants of inhibitions (*K*_i_) indicate that FA and 5OHFA in the active site of the LOX enzyme form a stable complex. The difference in energy of 1 kcal/mol in favour of the LOX-FA complex results in a six-time lower constant of inhibition, which means that FA has a higher affinity for the LOX enzyme. 

**Figure 5:**
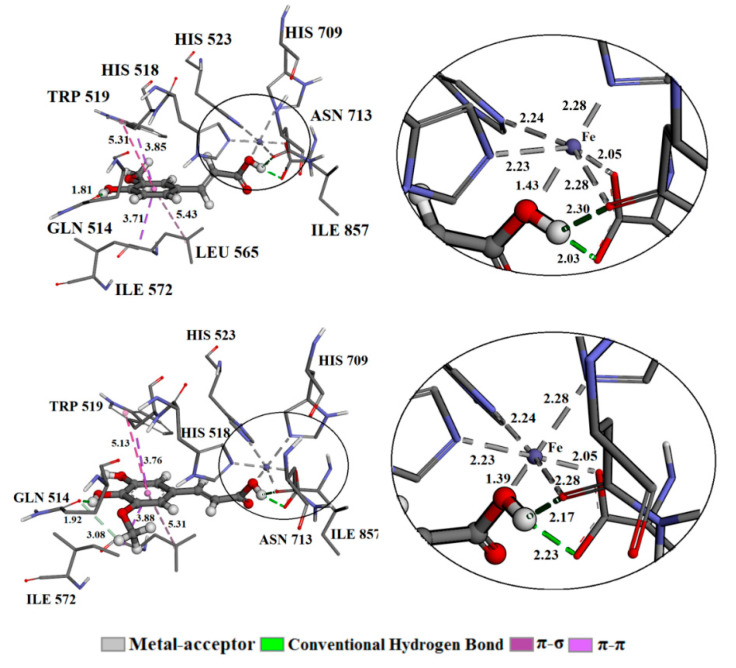
The best docking positions of FA (LOX-FA) and 5OHFA (LOX-5OHFA) compounds on the LOX enzyme.

An explanation of the difference in the thermodynamic parameters of the formed protein-ligand complexes can be found in the discussion of the binding mode of the investigated compounds in the active site of the enzyme. The most favourable compound orientations and discussions of established interactions offer a comprehensive mechanistic study of inhibitory activity. FA and 5OHFA in the LOX enzyme are presented in Figure 5. The types of interactions with the corresponding interatomic distances are summarized in the Table S2.

In the crystal structure, the active site of the enzyme is the iron ion surrounded by amino acid residues: HIS 518 (2.23 Å), HIS 523 (2.24 Å), HIS 709 (2.28 Å), ASN 713 (2.05 Å), and ILE 857 (2.28 Å), which directly participate in the catalytic mechanism of this enzyme. Three histidine (HIS) residues, via the nitrogen atom of the imidazole ring, establish metal-acceptor bonds, while the ASN and ILE do so via the oxygen atom of the carbonyl group. A 13-HPOD molecule, anchored at the enzyme site in the crystal structure, establishes a covalent complex at the sixth position with an iron ion via peroxy group [56]. Conversely, the analysis of the protein-ligand complex obtained after molecular docking simulation shows that, in both cases, the oxygen atom of the carboxyl group of FA and 5OHFA occupies the sixth position in the coordination sphere of iron, establishing a metal-acceptor bond. In both cases, a distorted octahedral geometry with *C*_3*v*_ symmetry is formed. The largest contribution to the total binding energy of LOX-FA and LOX-5OHFA comes from Δ*G*_elec_ (−6.41 and −5.30 kcal/mol), which is a consequence of these interactions.

A very important type of interaction that contributes to the stability of the protein-ligand complexes is the conventional hydrogen bond. The hydrogen atom of the hydroxyl group of FA and 5OHFA establishes bifurcated hydrogen bonding geometry with the oxygen of the carbonyl groups of the amino acids ASN 713 (2.03 and 2.23 Å, respectively) and ILE 857 (2.30 and 2.17 Å, respectively). As the mentioned amino acids also participate in the coordination with the iron ion, the established interactions will further destabilize the distorted octahedral (*C*_3*V*_) geometry. In addition, based on thermodynamic parameters, it is clear that the presence of an additional hydroxyl group on the 5OHFA molecule does not contribute to the binding affinity.

Conversely, OH groups attached to the aromatic ring of the FA and 5OHFA, responsible for antioxidant potency, established a conventional hydrogen bond with the oxygen atom of the amino acid GLN 514 (1.81 and 1.92 Å, respectively). This amino acid in the native enzyme participates in the creation of a series of hydrogen bonds that significantly stabilize the structure. In the crystal structure, GLN 514 is dislocated and its place is taken by C5−C6−C7 atoms from the 13-HPOD, which disrupts the network of hydrogen bonds and destabilizes the structure [56]. After molecular docking simulation, by establishing strong conventional hydrogen bonds, which are reflected in a small interatomic distance, FA and 5OHFA limit GLN 514 in stabilizing the protein structure. 

Also, the result presented in Figure 5 demonstrated that, in the active site, the investigated compounds are stabilized by several hydrophobic interactions. Both complexes are stabilized through the interaction of amino acid TRP 519 via the indole ring, which formed favorable π-σ and π-π contacts with the benzene ring of FA (3.85 and 5.31 Å, respectively) and 5OHFA (3.76 and 5.13 Å, respectively). Also, amino acid ILE 572, from both complexes, formed π-σ interaction with the benzene rings of FA (3.71 Å) and 5OHFA (3.88 Å). The molecular docking investigation justified the high antioxidant potency of the investigated compounds and their ability to provide direct and indirect antioxidant effects through LOX inhibition. Inhibition of prooxidant LOX enzyme, which requires much lower concentrations of antioxidants [39], could be assumed as a more contributing underlying mechanism of skin protection against UVA induced oxidative stress.

### 3.6. Chelation of Catalytic Fe^2+^ Ion

Liberation of iron ions in photodamaged skin may contribute to oxidative stress. As mentioned in the Section 1, in the presence of the iron catalyst, ^•^OH radicals may be generated in vivo via the Fenton reaction [7]:

H_2_O_2_ + Fe^2+^ → ^•^OH + ^−^OH + Fe^3+^ (2)

and Haber-Weiss reaction [14]:

(3) $$H_{2}O_{2}+{O_{2}}^{•-}\overset{Fe\left(II\right)/Fe\left(III\right)}{\to }{\;}^{•}\mathrm{OH}+{\;}^{-}\mathrm{OH}+O_{2}$$

Therefore, compounds able to chelate catalytic iron ions are expected to be effective in suppressing the ^•^OH production provided that complexation yields stable chelates (i.e., the complexation reactions should be exergonic). Iron chelators bind to Fe^2+^, rendering the cation inert. Fe^2+^ can coordinate six electron pair donor atoms of ligand(s) in an octahedral geometry. In the aqueous phase, hydrated [Fe(H_2_O)_6_]^2+^ ion exists, rather than ‘naked’ Fe^2+^ ion. This part of the investigation was performed in water as an ionization-supporting solvent, which enables the existence of involved ionic species.

Carboxylate group of FA is more acidic (p*K*_a1_ = 4.56) than its phenolic OH group (p*K*_a2_ = 8.65) [75]. It follows that at pH = 7.4, the molar fractions of neutral (AH), monoanionic (A^−^), and dianionic (A^2−^) species of FA are 0.0014, 0.9454, and 0.0532, respectively. Because there is no experimental p*K*_a_ values for 5OHFA, they were predicted by using the ACD/pKa GALAS algorithm [76] and amount to p*K*_a1_ = 4.4, p*K*_a2_ = 9.2, and p*K*_a3_ = 13.1. Related molar fractions are AH = 0.0010, A^−^ = 0.9834, and A^2−^ = 0.0156. Consequently, carboxylate anion of both acids (R-COO^−^) is the dominant active species and therefore is used as the main ligand in chelation of the Fe^2+^ ion; dianions are also considered ligands. Ferulate anion acts as a bidentate ligand, via its carboxylic oxygen atoms (or guaiacyl moiety oxygens), towards Fe^2+^. Recently, Fe^2+^/FA complexes with a 1:1 and 1:2 metal to ligand ratio have been described [77]. In this report, we considered both Fe^2+^/FA and Fe^2+^/5OHFA complexes with 1:1 and 1:2 metal to ligand molar ratios. As indicated by Holtomo and co-workers [78], the quintet spin state of Fe^2+^ was used as the most stable spin state.

The capacity of FA and 5OHFA to bound Fe^2+^ ions is evaluated from the Gibbs free energy of chelating reactions (Δ_r_*G*), as exemplified by the reaction between carboxylate anion of FA (FA^−^) with [Fe(H_2_O)_6_]^2+^:

FA^−^ + [Fe(H_2_O)_6_]^2+^ → [FAFe(H_2_O)_5_]^+^ + H_2_O (4)

Δ_r_*G* = *G*([FAFe(H_2_O)_5_]^+^) + *G*(H_2_O) − *G*(FA^−^) − *G*([Fe(H_2_O)_6_]^2+^) (5)

where *G*([FAFe(H_2_O)_5_]^+^), *G*(H_2_O), *G*(FA^−^), and *G*([Fe(H_2_O)_6_]^2+^) are the Gibbs free energy of complex, water, ferulate anion, and hydrated Fe^2+^ ion, respectively. 

Table 4 summarizes results obtained for the chelating reactions between the Fe^2+^ ion and monoanionic and dianionic forms of FA and 5OHFA in stoichiometric ratios of 1:1 and 1:2. The preferred coordination site for the Fe^2+^ ion are carboxylate oxygens of ferulate anion rather than the guaiacyl or catecholic oxygens, in line with the results of Truong and co-workers [77]. All studied complexation reactions in the aqueous phase at physiological pH = 7.4 are exergonic and spontaneous in terms of thermodynamics. The formation of bidentate complexes with a 1:2 ferrous ion to chelator ratio is more exergonic than complexes with a 1:1 ratio. The Δ_r_*G* values also indicate that 5OHFA demonstrates a slightly stronger chelation ability with the Fe^2+^ ion compared to FA in complexes with a 1:2 metal to ligand stoichiometric ratio. Thus, both FA and 5OHFA possess preventive antioxidant potential via the Fe^2+^ ion chelation, in such a way to be able to suppress its involvement in the Fenton and Haber-Weiss reactions.

Presented results for FA complexes are comparable with those recently published by Truong and co-workers [77]. The computational approach in this research differs from ours in it used M05 functional; we used M06 functional as a more appropriate following suggestion of developers of both functionals [53]. As demonstrated in Table S3, obtained Δ_r_*G* values by using M06 functional are less exergonic.

## 4. Conclusions

Little is known about the underlying mechanisms of skin protection by FA formulations from UVA-mediated free radicals damage. Besides mechanisms involving enzymes to suppress the formation of free radicals, the sequestration of transition metal ions that catalyse free radical production and direct scavenging could also be operative mechanisms if high concentrations of FA exist at the place of free radical formation. Performed kinetic analysis indicates that FA, 5OHFA, and their phenoxyl radicals effectively scavenge ^•^OH radicals with rate constants within the diffusion-controlled limit. Involved mechanisms between two free radicals (^•^OH and phenoxyl radical), which occur on the two PES via MECP, were specifically investigated. A reaction between FAPR and ^•^OH occurs via the RRC mechanism, while 5OHFAPR inactivates ^•^OH via the PCET mechanism. Keto-enol tautomerization, resulting in 5OHFA, proceeds via the hydrogen bond lattice of water molecules, leading to a high increase of reaction rate constant. Results of molecular docking simulations reveal that FA and 5OHFA form a stable complex in the active site of the LOX enzyme. Exergonicity of complexation reactions indicate FA and 5OHFA as potent chelators of ferrous ion.

Our results show that FA and 5OHFA have potential to act as multipotent antioxidants via direct free radical scavenging, suppressing ^•^OH radical formation by sequestering the catalytic Fe^2+^ ion, and by inhibiting the prooxidative LOX enzyme (i.e., by suppressing the formation of free radical precursors). Investigated compounds may show a synergistic effect in the antioxidant and inflammatory processes. The possible contribution of those mechanisms to skin protection in vivo is primarily determined by an achievable concentration of antioxidants in situ and in systemic circulation, which must be competitive with other endogenous scavengers and chelators. Inhibition of the prooxidant LOX enzyme, which require much lower concentrations of antioxidants, could be assumed as a more contributing mechanism. Further basic and clinical investigations are needed to determine the exact mechanism(s) underlying the skin protection efficacy of FA.

## Figures and Tables

**Table 1:** Reaction Gibbs free energy Δ_r_*G* (kcal/mol), activation Gibbs free energy Δ*G*^≠^ (kcal/mol), MECP imaginary frequency *ν* (cm^−1^) in singlet state, Eckart tunneling coefficient *κ* ^Eck^, *k*^TST^, and *k*^TST/Eck^ rate constant (M^−1^ s^−1^) at 298.15 K, in RRC pathways of FAPR with the ^•^OH radical.

Path	Δ_r_*G*	Δ*G*^≠^	*ν*	*k* ^TST^	*κ* ^Eck^	*k* ^TST/Eck^
C1	−41.5	14.1	−256	2.71 × 10^2^	1.1	2.89 × 10^2^
C3	−48.2	11.1	−254	4.70 × 10^4^	1.1	5.00 × 10^4^
C5	−47.6	12.9	−363	2.23 × 10^3^	1.1	2.53 × 10^3^
C8	−50.4	8.6	−306	3.31 × 10^6^	0.9	2.98 × 10^6^
$k_{\mathrm{overall}}^{\mathrm{TST}/\mathrm{Eck}}$=						3.03 × 10^6^

**Table 2:** Number of water molecules *n*, reaction Gibbs free energy Δ_r_*G* (kcal/mol), activation Gibbs free energy Δ*G*^≠^ (kcal/mol), imaginary frequency *ν* (cm^−1^), Eckart tunneling coefficient *κ* ^Eck^, *k*^TST^, and *k*^TST/Eck^ rate constant (s^−1^) at 298.15 K.

*n*	Δ_r_*G*	Δ*G*^≠^	*ν*	*k* ^TST^	*κ* ^Eck^	*k* ^TST/Eck^
0	−28.2	72.9	−1551	2.3 × 10^−41^	1282.7	3.0 × 10^−38^
1	−24.3	23.9	−1384	1.9 × 10^−5^	10.5	2.0 × 10^−4^
2	−27.1	11.8	−966	1.4 × 10^4^	2.8	4.1 × 10^4^
3	−27.2	10.1	−838	2.3 × 10^5^	2.1	4.8 × 10^5^

**Table 3:** Estimated important thermodynamic parameters in kcal/mol (Δ*G*_bind_ free energy binding, *K*_i_ constant of inhibition, Δ*G*_total_ final total internal energy, Δ*G*_tor_ torsional free energy, Δ*G*_unb_ unbound system’s energy, Δ*G*_elec_ electrostatic energy, and Δ*G*_vdw+hbond+desolv_ is the sum of the effect of dispersion and repulsion (Δ*G*_vdw_), hydrogen bond (Δ*G*_hbond_), and desolvation (Δ*G*_desolv_)) obtained after the molecular docking simulation. The intermolecular energy, Δ*G*_inter_, represents the sum of Δ*G*_vdw+hbond+desolv_ and Δ*G*_elec_.

Conformation	Δ*G*_bind_	*K*_i_ (µM)	Δ*G*_inte*r*_	Δ*G*_vdw+hbond+desolv_	Δ*G*_elec_	Δ*G*_total_	Δ*G*_tor_	Δ*G*_unb_
LOX-FA	−8.29	0.84	−9.78	−3.37	−6.41	−0.25	1.49	−0.25
LOX-5OHFA	−7.25	4.82	−9.04	−3.75	−5.30	−1.72	1.79	−1.72

**Table 4:** Structures of complexes and reaction Gibbs free energies (Δ_r_*G*) of the complexation reactions between the Fe^2+^ ion with FA^−^ (FA^2−^) and 5OHFA^−^ (5OHFA^2−^) in stoichiometric ratios of 1:1 and 1:2.

1:1 Fe^2+^−FA Complexes	1:1 Fe^2+^−5OHFA Complexes
Fe^2+^−FA^−^ 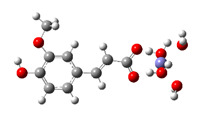 Δ_r_*G* = −9.2476 kcal/mol	Fe^2+^−5OHFA^−^ 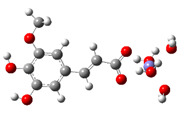 Δ_r_*G* = −9.8757 kcal/mol
Fe^2+^−FA^2−^ 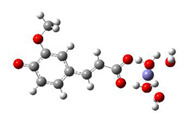 Δ_r_*G* = −12.3638 kcal/mol	Fe^2+^−5OHFA^2−^ 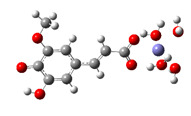 Δ_r_*G* = −11.2186 kcal/mol
Fe^2+^−FA^−^ 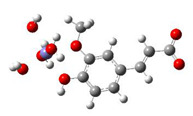 Δ_r_*G* = −2.5853 kcal/mol	Fe^2+^−5OHFA^−^ 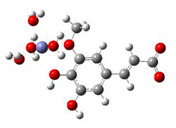 Δ_r_*G* = −1.8091 kcal/mol
Fe^2+^−FA^2−^ 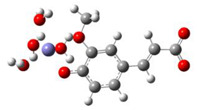 Δ_r_*G* = −16.4640 kcal/mol	Fe^2+^−FA^2−^ 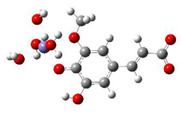 Δ_r_*G* = −16.2287 kcal/mol
1:2 Fe^2+^−FA complexes	1:2 Fe^2+^−5OHFA complexes
Fe^2+^−(FA^−^)_2_ 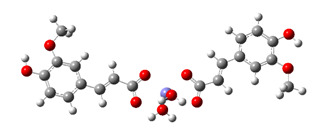 Δ_r_*G* = −20.9369 kcal/mol	Fe^2+^−(5OHFA^−^)_2_ 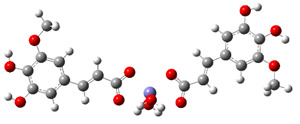 Δ_r_*G* = −21.7620 kcal/mol
Fe^2+^−(FA^2−^)_2_ 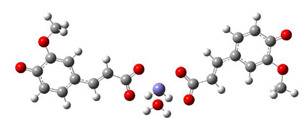 Δ_r_*G* = −23.9257 kcal/mol	Fe^2+^−(5OHFA^2−^)_2_ 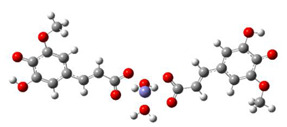 Δ_r_*G* = −25.6670 kcal/mol

## Data Availability

Data is contained within the article and supplementary material.

## Supplementary Materials

The following are available online at https://www.mdpi.com/article/10.3390/antiox10081303/s1: Table S1: Reaction Gibbs free energy (Δ_r_*G*, kcal/mol), activation Gibbs free energy (Δ*G*^≠^, kcal/mol), TS imaginary frequency (*ν*, cm^−1^), TST rate constants (*k*^TST^ and *k*^TST/Eck^, M^−1^ s^−1^), and Eckart tunneling coefficient (*κ*^Eck^) in the H-atom abstraction from: (a) 4-OH group of FA, (b) 4-OH group of 5OHFA, and (c) 5-OH group of 5OHFAPR by ^•^OH radical at 298.15 K in gas-phase; Figure S1: Optimized geometries obtained in gas-phase with the M06-2X/6-311++G(d,p) level of theory in the reaction of ^•^OH radical with 4-OH group of FA; Figure S2: Energy profiles for RRC pathways of C5 site of FAPR with ^•^OH in the singlet (red line) and triplet (black line) states, created using a published procedure [51]; Figure S3: Activation Gibbs free energy (Δ*G*^≠^ in kcal/mol) as a function of the number of water molecules; Figure S4: Reaction rate constant (*k*^TST/Eck^ in M^−1^ s^−1^) as a function of the number of water molecules; Figure S5: Optimized structures involved in keto-enol tautomerization aided by three catalytic water molecules; Figure S6: Optimized geometries obtained in gas-phase with the M06-2X/6-311++G(d,p) level of theory in the reaction of ^•^OH radical with: a) 4-OH group of 5OHFA, and b) 5-OH group of phenoxyl radical of 5-hydroxyferulic acid (5OHFAPR); Table S2: Type of interactions and corresponding distance (Å) of most stable docking structures LOX-FA and LOX-5OHFA; Table S3: Structures and Gibbs free energies Δ_r_*G* (kcal/mol) of the complexation reactions between Fe^2+^ and FA. Comparison of results published by Troung and co-workers [77] with those presented in this work. Optimized geometries and Cartesian coordinates of TSs in PCET and RRC reactions, TSs in keto-enol tautomerization, and iron chelates.
