# Supplementary material for: Theoretical Study of Radical Inactivation, LOX Inhibition, and Iron Chelation: The Role of Ferulic Acid in Skin Protection against UVA Induced Oxidative Stress
Source: Antioxidants (Basel). 2021 Aug 18;10(8):1303. doi: 10.3390/antiox10081303 (PMC8389219; doi:10.3390/antiox10081303)
Supplement: Supplementary file 1 [file antioxidants-10-01303-s001.zip › antioxidants-1325839-supplementary.pdf]

Supporting Information

Theoretical study of radical inactivation, LOX inhibition and iron chelation: a role of ferulic acid in skin protection against UVA induced oxidative stress

Ana Amić[1*], Jasmina M. Dimitrić Marković[2], Zoran Marković[3], Dejan Milenković[3], Žiko Milanović[3,4], Marko Antonijević[3], Denisa Mastiľák Cagardová[5], Jaime Rodríguez-Guerra Pedregal[6]

[1] Department of Chemistry, Josip Juraj Strossmayer University of Osijek, Ulica cara Hadrijana 8A, 31000 Osijek, Croatia
e-mail: aamic@kemija.unios.hr

[2] The Faculty of Physical Chemistry, University of Belgrade, Studentski trg 12-16, 11000 Belgrade, Serbia
e-mail: markovich@ffh.bg.ac.rs

[3] Institute for Information Technologies, Department of Science, University of Kragujevac, 34000 Kragujevac, Serbia
e-mail: zmarkovic@uni.kg.ac.rs; deki82@kg.ac.rs; mantonijevic@uni.kg.ac.rs

[4] Faculty of Science, Department of Chemistry, University of Kragujevac, Radoja Domanovića 2, 34000 Kragujevac, Serbia
e-mail: ziko.milanovic@uni.kg.ac.rs

[5] Institute of Physical Chemistry and Chemical Physics, Department of Chemical Physics, Slovak University of Technology in Bratislava, Radlinského 9, SK-812 37 Bratislava, Slovak Republic
e-mail: denisa.cagardova@stuba.sk

[6] In Silico Toxicology, Institute of Physiology, Charité - Universitätsmedizin Berlin, Charitéplatz 1, 10117 Berlin, Germany
e-mail: jaime.rodriguez@charite.de

**corresponding author*

Table S1. Reaction Gibbs free energy ($\Delta_r G$, kcal/mol), activation Gibbs free energy ($\Delta G^{\neq}$, kcal/mol), TS imaginary frequency (ν, cm^{-1}), TST rate constants (k^{TST} and $k^{TST/Eck}$, M^{-1} s^{-1}) and Eckart tunneling coefficient (κ^{Eck}) in the H-atom abstraction from: a) 4-OH group of FA, b) 4-OH group of 5OHFA, and c) 5-OH group of 5OHFAPR by $^{\bullet}$OH radical at 298.15 K in gas-phase.

	path	$\Delta_r G$	$\Delta G^{\neq}$	ν	k^{TST}	κ^{Eck}	$k^{TST/Eck}$
a	4-OH	-29.9	6.5	-2347	1.08×10^8	21.4	2.29×10^9
b	4-OH	-36.8	4.6	-2872	2.53×10^9	8.3	2.11×10^{10}
c	5-OH	-43.1	4.7	-1163	2.29×10^9	3.3	7.83×10^9

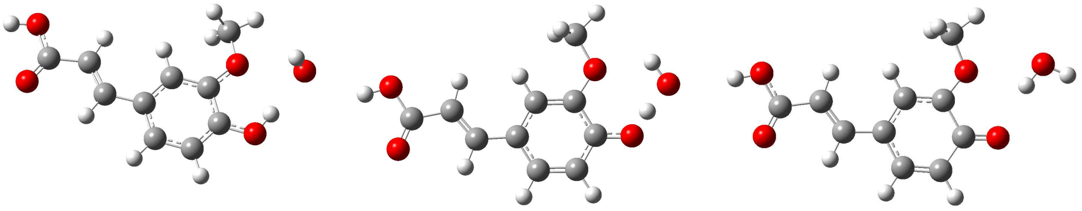

RC TS PC

Figure S1. Optimized geometries obtained in gas-phase with the M06-2X/6-311++G(d,p) level of theory in the reaction of $^{\bullet}$OH radical with 4-OH group of FA.

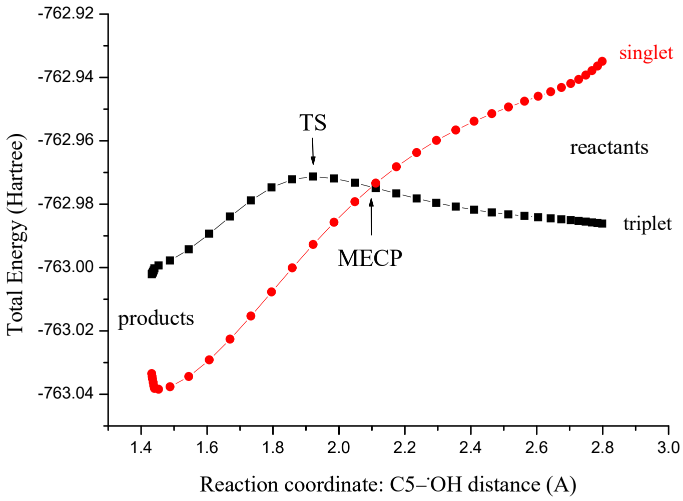

Figure S2. Energy profiles for RRC pathways of C5 site of FAPR with $^{\bullet}$OH in the singlet (red line) and triplet (black line) states, created using published procedure [51].

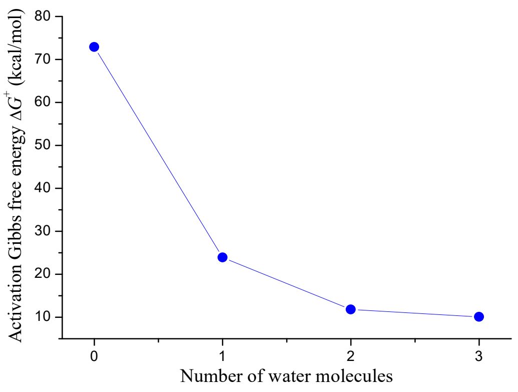

Figure S3. Activation Gibbs free energy ($\Delta G^{\neq}$ in kcal/mol) as a function of the number of water molecules.

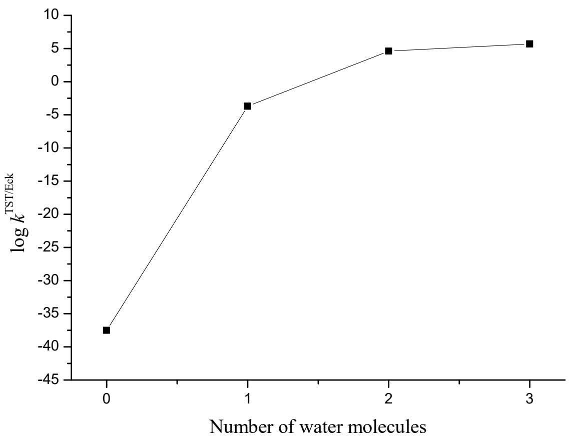

Figure S4. Reaction rate constant ($k^{\mathrm{TST/Eck}}$ in M^{-1} s^{-1}) as a function of the number of water molecules.

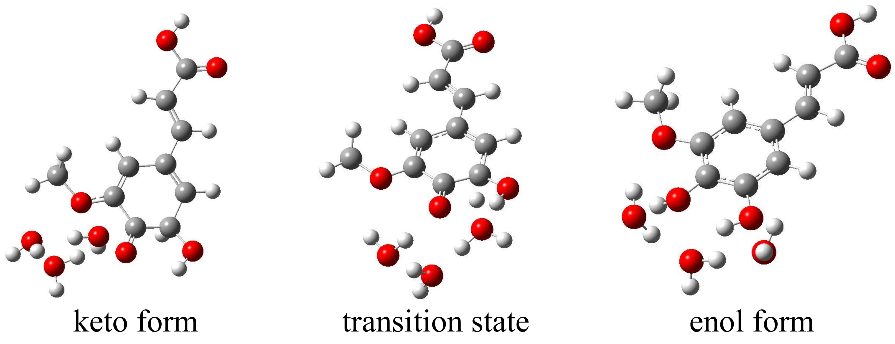

Figure S5. Optimized structures involved in keto-enol tautomerization aided by three catalytic water molecules.

a Inactivation of $^{\bullet}$OH radical by 4-OH group of 5OHFA

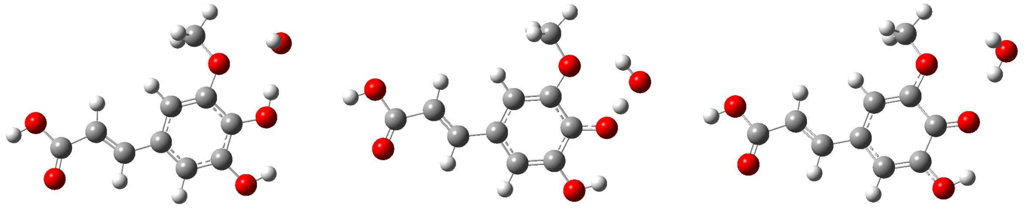

RC TS PC

b Inactivation of $^{\bullet}$OH radical by 5-OH group of 5OHFAPR

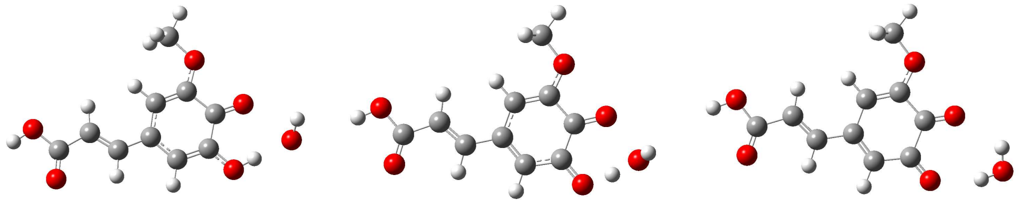

RC in triplet TS in triplet PC in singlet

Figure S6. Optimized geometries obtained in gas-phase with the M06-2X/6-311++G(d,p) level of theory in the reaction of $^{\bullet}$OH radical with: a) 4-OH group of 5OHFA, and b) 5-OH group of phenoxyl radical of 5-hydroxyferulic acid (5OHFAPR).

Table S2. Type of interactions and corresponding distance (Å) of most stable docking structures LOX-FA and LOX-5OHFA

Conformation of the ligand	Interaction	Category	Type of interaction	Distance (Å)
**LOX-FA**	Fe - A:HIS518:N	Electrostatic	Metal-Acceptor	2.23
	Fe - A:HIS523:N	Electrostatic	Metal-Acceptor	2.24
	Fe - A:HIS709:N	Electrostatic	Metal-Acceptor	2.28
	Fe - A:ASN713:O	Electrostatic	Metal-Acceptor	2.28
	Fe - A:ILE857:O	Electrostatic	Metal-Acceptor	2.05
	Fe - FA:OH	Electrostatic	Metal-Acceptor	1.43
	FA:H - A:ASN713:O	Hydrogen Bond	Conventional Hydrogen Bond	2.30
	FA:H - A:ILE857:O	Hydrogen Bond	Conventional Hydrogen Bond	2.03
	FA:H - A:GLN514:O	Hydrogen Bond	Conventional Hydrogen Bond	1.81
	A:TRP519:C - FA	Hydrophobic	Pi-Sigma	3.85
	A:ILE572:C - FA	Hydrophobic	Pi-Sigma	3.70
	A:TRP519 - FA	Hydrophobic	Pi-Pi Stacked	5.31
	FA - A:LEU565	Hydrophobic	Pi-Alkyl	5.43
**LOX-5OHFA**	Fe - A:HIS518:N	Electrostatic	Metal-Acceptor	2.23
	Fe - A:HIS523:N	Electrostatic	Metal-Acceptor	2.24
	Fe - A:HIS709:N	Electrostatic	Metal-Acceptor	2.28
	Fe - A:ASN713:O	Electrostatic	Metal-Acceptor	2.28
	Fe - A:ILE857:O	Electrostatic	Metal-Acceptor	2.05
	Fe – 5OHFA:OH	Electrostatic	Metal-Acceptor	1.39
	5OHFA:H - A:ASN713:O	Hydrogen Bond	Conventional Hydrogen Bond	2.17
	5OHFA:H - A:ILE857:O	Hydrogen Bond	Conventional Hydrogen Bond	2.23
	5OHFA:H - A:GLN514:O	Hydrogen Bond	Conventional Hydrogen Bond	1.92
	A:TRP519:C – 5OHFA	Hydrophobic	Pi-Sigma	3.76
	A:ILE572:C – 5OHFA	Hydrophobic	Pi-Sigma	3.88
	A:TRP519 – 5OHFA	Hydrophobic	Pi-Pi Stacked	5.13
	5OHFA - A:LEU565	Hydrophobic	Pi-Alkyl	5.31

Table S3. Structures and Gibbs free energies $\Delta_r G$ (kcal/mol) of the complexation reactions between Fe^{2+} and FA. Comparison of results published by Truong and co-workers [77] with those presented in this work.

	Ref. 77	our result
1:1 Fe^{2+}–FA	$\Delta_r G$ (kcal/mol)	$\Delta_r G$ (kcal/mol)
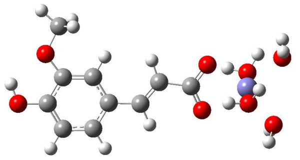	-20.6	-9.25
1:2 Fe^{2+}–FA		
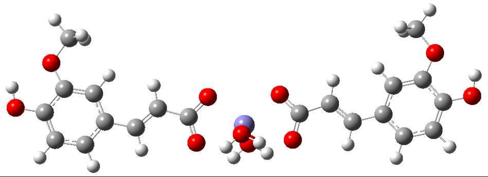	-45.3	-20.55

Truong and co-workers [77] used SMD/M05/6-311++g(d,p) level of theory in water as a solvent. Authors were not consider dianions of ferulic acid as chelators. Our results were obtained by using SMD/M06/6-311++g(d,p) level of theory in water as a solvent.

Optimized geometries and corresponding Cartesian coordinates

M06-2X/6-311++G(d,p) optimized geometry of TS for the reaction of 4-OH group of ferulic acid with $^{\bullet}$OH radical in gas-phase.

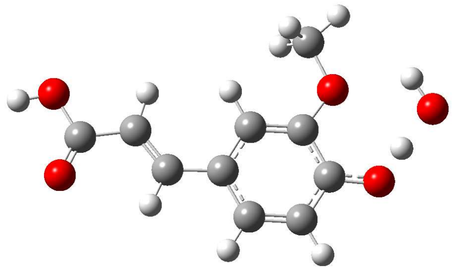

6	2.230897000	-0.997618000	-0.173110000
6	1.600336000	0.277140000	-0.157016000
6	0.223867000	0.375918000	-0.075441000
6	-0.572433000	-0.779036000	-0.021403000
6	0.052031000	-2.032904000	-0.028366000
6	1.427853000	-2.140391000	-0.090599000
8	3.534337000	-1.138992000	-0.291104000
1	4.148082000	-0.340237000	-0.005246000
8	4.936258000	0.511342000	0.740739000
1	1.926022000	-3.101583000	-0.091734000
1	-0.557329000	-2.928194000	0.017184000
1	-0.247647000	1.348622000	-0.055372000
8	2.450478000	1.333285000	-0.201638000
6	1.886847000	2.635147000	-0.216369000
1	1.318733000	2.825476000	0.698302000
1	2.724317000	3.326550000	-0.279083000
1	1.242524000	2.765228000	-1.089844000
6	-2.028775000	-0.721344000	0.046207000
6	-2.801559000	0.370653000	-0.001878000
1	-2.541139000	-1.676212000	0.143091000
1	-2.418870000	1.377828000	-0.106676000
6	-4.267323000	0.223868000	0.085670000
8	-4.887541000	1.420921000	0.016316000
8	-4.872013000	-0.809484000	0.203803000
1	-5.837102000	1.253420000	0.079260000
1	4.439813000	1.343922000	0.739007000

M06-2X/6-311++G(d,p) optimized geometry of TS (in triplet) for the reaction of C5 site of ferulic acid phenoxyl radical with •OH radical in gas-phase.

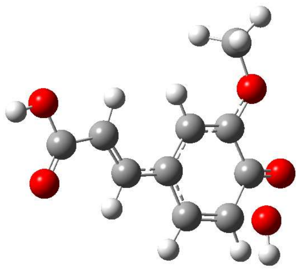

6	1.880599000	0.730294000	-0.119034000
6	2.693490000	-0.471309000	-0.378069000
6	1.943731000	-1.735015000	-0.370920000
6	0.561470000	-1.726797000	-0.459431000
6	-0.198570000	-0.518257000	-0.226876000
6	0.484051000	0.675369000	-0.047001000
1	2.514385000	-2.607398000	-0.664227000
1	0.022041000	-2.650872000	-0.635362000
1	-0.075197000	1.582903000	0.136465000
8	3.897881000	-0.414002000	-0.563256000
8	2.592683000	1.834954000	0.014494000
6	1.931885000	3.062940000	0.283660000
1	1.371769000	3.001341000	1.219946000
1	2.719219000	3.805913000	0.374447000
1	1.264145000	3.329857000	-0.539469000
6	-1.640004000	-0.619436000	-0.209484000
6	-2.521699000	0.375541000	-0.003985000
1	-2.060248000	-1.608750000	-0.376248000
1	-2.239898000	1.404490000	0.179857000
6	-3.965791000	0.074632000	-0.019464000
8	-4.460136000	-1.006645000	-0.205657000
8	-4.702392000	1.181823000	0.205095000
1	-5.631654000	0.917205000	0.182483000
8	1.830021000	-2.007239000	1.528546000
1	1.836006000	-2.973582000	1.591956000

M06-2X/6-311++G(d,p) geometry of MECP (in singlet) for the reaction of C5 site of ferulic acid phenoxyl radical with $^{\bullet}$OH radical in gas-phase.

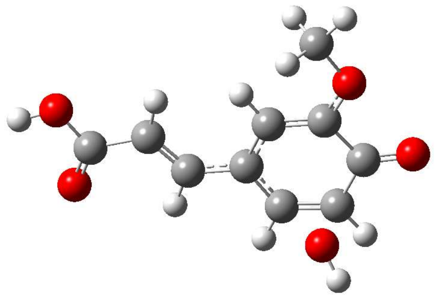

6	1.856157000	0.705950000	-0.156301000
6	2.661720000	-0.516360000	-0.437341000
6	1.898508000	-1.769468000	-0.467100000
6	0.527037000	-1.772020000	-0.452266000
6	-0.202401000	-0.574089000	-0.248867000
6	0.485720000	0.651478000	-0.089181000
1	2.471862000	-2.668053000	-0.662995000
1	-0.017435000	-2.703948000	-0.553515000
1	-0.081580000	1.553603000	0.093049000
8	3.859494000	-0.466208000	-0.627660000
8	2.593784000	1.796634000	-0.018506000
6	1.939819000	3.022059000	0.268104000
1	1.368794000	2.942479000	1.196797000
1	2.729743000	3.758715000	0.384153000
1	1.279484000	3.309444000	-0.555304000
6	-1.646006000	-0.661182000	-0.205246000
6	-2.509421000	0.353897000	-0.030199000
1	-2.077060000	-1.651834000	-0.327369000
1	-2.209434000	1.385084000	0.103290000
6	-3.960042000	0.076341000	-0.011928000
8	-4.473815000	-1.002503000	-0.150073000
8	-4.673747000	1.203504000	0.182409000
1	-5.607996000	0.955284000	0.185886000
8	1.894013000	-1.703854000	1.766869000
1	2.239236000	-2.600995000	1.893645000

M06-2X/6-311++G(d,p) optimized geometry of TS for the reaction of 4-OH group of 5-hydroxyferulic acid with •OH radical in gas-phase.

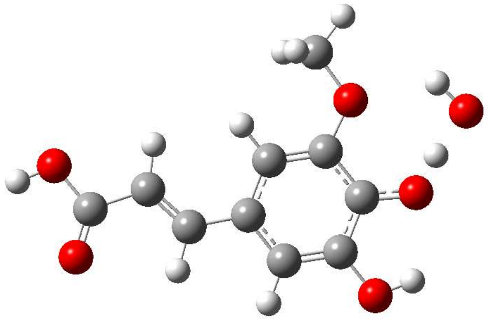

6	2.116124000	-0.624195000	-0.143772000
6	1.413195000	0.599461000	-0.126731000
6	0.031172000	0.612935000	-0.055164000
6	-0.680213000	-0.597359000	-0.004489000
6	0.008356000	-1.812181000	-0.005622000
6	1.391500000	-1.826811000	-0.067845000
8	3.434061000	-0.735145000	-0.239249000
1	4.011985000	0.088585000	0.016721000
8	4.802276000	1.063810000	0.656096000
1	-0.523182000	-2.754686000	0.042068000
1	-0.503745000	1.551102000	-0.031809000
8	2.202037000	1.707084000	-0.161726000
6	1.554579000	2.968972000	-0.216131000
1	0.966142000	3.145180000	0.688277000
1	2.345191000	3.712633000	-0.290292000
1	0.911975000	3.032044000	-1.097946000
6	-2.140162000	-0.635575000	0.057788000
6	-2.979568000	0.403727000	-0.013646000
1	-2.589532000	-1.619712000	0.170947000
1	-2.660317000	1.430362000	-0.138409000
6	-4.434243000	0.167774000	0.074963000
8	-5.126676000	1.322974000	-0.016527000
8	-4.974184000	-0.898468000	0.211688000
1	-6.064248000	1.098677000	0.049684000
1	4.218179000	1.839258000	0.634011000
8	2.061912000	-2.993985000	-0.068203000
1	3.007714000	-2.794095000	-0.095978000

M06-2X/6-311++G(d,p) optimized geometry of TS (in triplet) for the reaction of 5-OH group of phenoxyl radical of 5-hydroxyferulic acid with •OH radical in gas-phase.

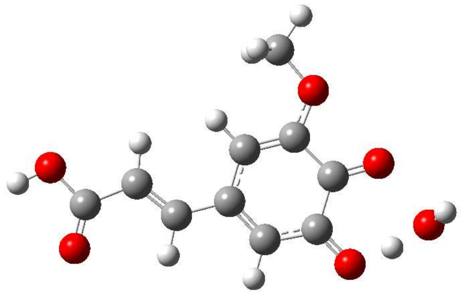

6	2.240151000	0.054275000	0.005995000
6	1.272059000	1.141691000	0.009422000
6	-0.066061000	0.877033000	0.006952000
6	-0.542063000	-0.472937000	0.009393000
6	0.338699000	-1.529283000	0.017442000
6	1.738513000	-1.325257000	0.014811000
8	3.469684000	0.338980000	0.006988000
8	4.726308000	-1.353787000	-0.160323000
1	-0.011811000	-2.553958000	0.022942000
1	-0.776738000	1.690024000	0.003836000
8	1.839026000	2.357014000	0.013747000
6	0.976058000	3.478735000	-0.001434000
1	0.339218000	3.491501000	0.888191000
1	1.622354000	4.352425000	-0.002367000
1	0.354382000	3.478409000	-0.901713000
6	-1.972607000	-0.767892000	0.007037000
6	-2.973209000	0.120277000	-0.007880000
1	-2.246455000	-1.820241000	0.017746000
1	-2.831224000	1.192991000	-0.021276000
6	-4.371491000	-0.361810000	-0.007343000
8	-5.243581000	0.666237000	-0.026013000
8	-4.723104000	-1.511212000	0.007587000
1	-6.133122000	0.288647000	-0.025272000
1	5.100405000	-1.075398000	0.685395000
8	2.561027000	-2.299799000	0.036768000
1	3.707822000	-1.912881000	-0.023889000

M06-2X/6-311++G(d,p) optimized geometry of TS for keto-enol tautomerization of 5-hydroxyferulic acid in gas-phase.

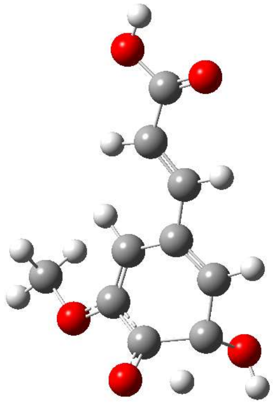

6	-0.442083000	0.700049000	-0.101664000
6	-1.762420000	0.918057000	0.055025000
6	-2.440672000	-0.281013000	0.499243000
6	-2.097525000	-1.702746000	-0.031514000
6	-0.623881000	-1.779406000	-0.053358000
6	0.144976000	-0.654213000	-0.015948000
1	0.230125000	1.529421000	-0.285649000
1	-0.163703000	-2.750425000	-0.193789000
8	-3.135158000	-0.437858000	1.577122000
8	-2.466225000	2.051797000	-0.066581000
1	-3.036639000	-1.701157000	0.985031000
6	-1.745254000	3.189887000	-0.543739000
1	-1.261332000	2.965924000	-1.507089000
1	-2.475742000	3.990138000	-0.678706000
1	-0.992514000	3.515725000	0.197393000
8	-2.652066000	-2.108357000	-1.296389000
1	-3.424954000	-2.661671000	-1.108750000
6	1.600696000	-0.768874000	0.010191000
6	2.488467000	0.243055000	0.061362000
1	2.004527000	-1.772325000	-0.011371000
1	2.214016000	1.283349000	0.111460000
6	3.937483000	-0.044531000	0.063916000
8	4.456319000	-1.127657000	0.062654000
8	4.666763000	1.101188000	0.067395000
1	5.590441000	0.846525000	0.076780000

M06-2X/6-311++G(d,p) optimized geometry of TS for keto-enol tautomerization of 5-hydroxyferulic acid aided by one water molecule in gas-phase.

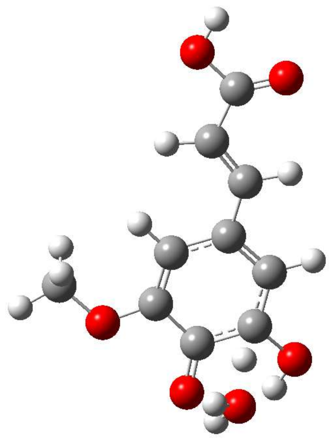

6	0.491050000	-0.455578000	-0.202754000
6	-0.068421000	0.855366000	-0.121361000
6	-1.418833000	1.073870000	-0.204783000
6	-2.344331000	-0.029121000	-0.405558000
6	-0.348401000	-1.531968000	-0.383281000
1	0.599787000	1.693656000	0.022782000
1	-2.156732000	-1.174190000	1.003952000
8	-3.601021000	0.077046000	-0.390514000
8	-3.062962000	-1.169752000	1.857219000
1	-3.699344000	-0.685448000	1.263174000
1	-2.837001000	-0.583681000	2.589718000
6	-1.748660000	-1.339566000	-0.410025000
8	-2.570514000	-2.390154000	-0.744944000
1	-3.423732000	-1.996753000	-0.982697000
1	0.039028000	-2.539149000	-0.488960000
8	-2.025711000	2.275771000	-0.132685000
6	-1.198950000	3.418015000	-0.051573000
1	-0.528954000	3.477615000	-0.915181000
1	-1.868022000	4.274977000	-0.048333000
1	-0.605902000	3.413220000	0.869065000
6	1.926674000	-0.689930000	-0.114816000
1	2.245228000	-1.728396000	-0.179433000
6	2.893852000	0.224500000	0.034593000
1	2.713780000	1.289281000	0.105401000
6	4.298121000	-0.214756000	0.102702000
8	5.138733000	0.834411000	0.242594000
1	6.034775000	0.475013000	0.276948000
8	4.695786000	-1.349964000	0.046418000

M06-2X/6-311++G(d,p) optimized geometry of TS for keto-enol tautomerization of 5-hydroxyferulic acid aided by two water molecules in gas-phase.

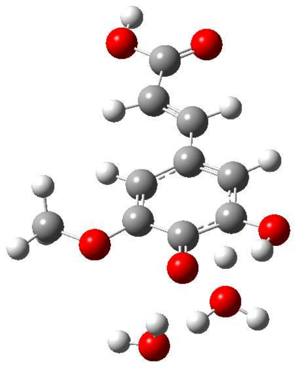

6	-0.794577000	-0.513510000	-0.273451000
6	-0.153599000	0.767691000	-0.261713000
6	1.190513000	0.907245000	-0.462197000
6	2.031531000	-0.248998000	-0.750560000
6	1.386435000	-1.525934000	-0.554726000
6	-0.028027000	-1.637717000	-0.460679000
1	-0.759466000	1.644592000	-0.074674000
8	3.257125000	-0.192596000	-0.980163000
1	1.659393000	-1.292719000	0.769265000
8	3.855625000	0.729564000	1.475323000
1	3.909127000	0.597403000	0.504167000
1	3.551230000	1.634591000	1.585719000
8	2.187015000	-1.193943000	1.968722000
1	2.861563000	-0.446060000	1.903804000
1	2.647405000	-2.001657000	2.221184000
8	1.872968000	2.077483000	-0.462767000
1	-0.470932000	-2.627473000	-0.482390000
8	2.137940000	-2.639756000	-0.866043000
1	2.998247000	-2.297373000	-1.154502000
6	1.115932000	3.270238000	-0.389081000
1	1.828307000	4.086795000	-0.481271000
1	0.391782000	3.320705000	-1.207777000
1	0.590346000	3.347486000	0.568376000
6	-2.233370000	-0.657272000	-0.099866000
1	-2.612480000	-1.677045000	-0.089265000
6	-3.138827000	0.319686000	0.040585000
1	-2.896636000	1.374583000	0.037338000
6	-4.559846000	-0.031841000	0.203718000
8	-5.022107000	-1.143198000	0.235600000
8	-5.332438000	1.071270000	0.320026000
1	-6.243903000	0.768045000	0.422265000

M06-2X/6-311++G(d,p) optimized geometry of TS for keto-enol tautomerization of 5-hydroxyferulic acid aided by three water molecules in gas-phase.

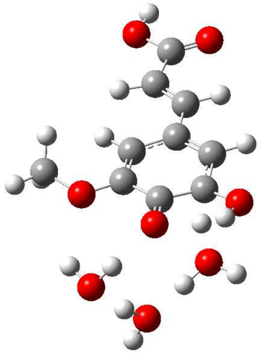

6	-1.141324000	-0.479951000	-0.389583000
6	-0.456265000	0.778436000	-0.306390000
6	0.879494000	0.900583000	-0.555020000
6	1.676491000	-0.258100000	-0.950533000
6	0.995593000	-1.531403000	-0.832664000
6	-0.423348000	-1.602617000	-0.711312000
1	-1.031812000	1.658250000	-0.050024000
8	2.875222000	-0.223701000	-1.279196000
1	1.284189000	-1.559680000	0.471235000
8	4.249738000	1.411684000	0.378294000
1	3.966840000	0.863370000	-0.382323000
1	3.602171000	2.123237000	0.350515000
8	1.795435000	-1.915373000	1.652958000
1	2.527665000	-1.283986000	1.942219000
1	2.195391000	-2.783681000	1.531772000
8	1.573687000	2.068066000	-0.555631000
1	-0.900180000	-2.572607000	-0.803583000
8	1.684468000	-2.627938000	-1.323079000
1	2.539150000	-2.281410000	-1.622668000
6	0.829239000	3.266771000	-0.434863000
1	1.542335000	4.079605000	-0.554376000
1	0.067996000	3.328783000	-1.218016000
1	0.352958000	3.339300000	0.547760000
6	-2.573800000	-0.593027000	-0.155834000
1	-2.996704000	-1.589668000	-0.263716000
6	-3.425956000	0.386562000	0.174454000
1	-3.135658000	1.419486000	0.315171000
6	-4.851271000	0.068923000	0.365623000
8	-5.363214000	-1.015477000	0.254681000
8	-5.563991000	1.169362000	0.694426000
1	-6.481760000	0.888620000	0.803967000
8	3.693578000	-0.320183000	2.291653000
1	3.890594000	0.014191000	3.168014000
1	3.964332000	0.367620000	1.627926000

M06/6-311++G(d,p) optimized geometry of $[Fe(H_2O)_6]^{2+}$ in water.

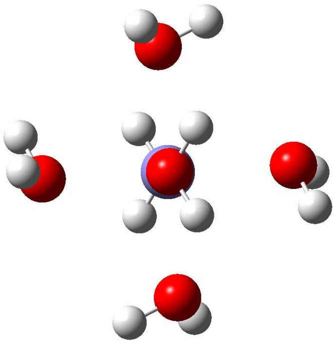

26	0.000000000	0.000000000	0.000000000
8	-1.824913000	-0.516134000	-1.124891000
8	1.193712000	-0.940927000	-1.603175000
1	2.138018000	-0.758932000	-1.525287000
1	1.118512000	-1.902485000	-1.584923000
8	-1.193712000	0.940927000	1.603175000
1	-2.138018000	0.758932000	1.525287000
1	-1.118512000	1.902485000	1.584923000
8	-0.105964000	-1.898623000	1.096580000
1	0.707502000	-2.409272000	1.000422000
1	-0.204521000	-1.753639000	2.045580000
8	0.105964000	1.898623000	-1.096580000
1	0.204521000	1.753639000	-2.045580000
1	-0.707502000	2.409272000	-1.000422000
8	1.824913000	0.516134000	1.124891000
1	1.654363000	0.483296000	2.074546000
1	2.069493000	1.432180000	0.943396000
1	-1.654363000	-0.483296000	-2.074546000
1	-2.069493000	-1.432180000	-0.943396000

M06/6-311++G(d,p) optimized geometry of 1:1 Fe^{2+}−FA complex in water.

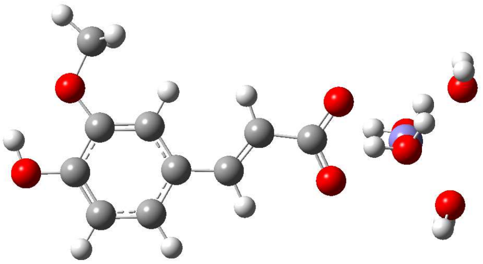

6	-3.304443000	0.460805000	0.012007000
6	-4.682967000	0.431072000	0.017733000
6	-5.362435000	-0.801620000	0.003414000
6	-4.648030000	-1.982921000	-0.022051000
6	-3.260613000	-1.950121000	-0.033406000
6	-2.570465000	-0.737973000	-0.013048000
6	-1.118357000	-0.763602000	-0.022113000
6	-0.277826000	0.277872000	0.052776000
6	1.180506000	0.100088000	0.029037000
6	-4.900553000	2.799774000	-0.026400000
8	-5.500989000	1.515570000	0.032972000
8	1.705133000	-1.038883000	-0.098453000
8	1.916877000	1.137992000	0.139385000
8	-6.718953000	-0.833114000	0.009512000
1	-0.676386000	-1.757744000	-0.097527000
1	-0.631055000	1.302355000	0.141143000
1	-2.698792000	-2.879640000	-0.056648000
1	-5.191010000	-2.922803000	-0.037078000
1	-2.785214000	1.413383000	0.022196000
1	-4.309266000	2.911962000	-0.941482000
1	-5.717193000	3.520881000	-0.031103000
1	-4.264623000	2.977315000	0.847258000
1	-7.053461000	0.076016000	0.015302000
26	3.764643000	0.050998000	-0.004867000
8	3.798480000	-0.179809000	2.216857000
8	4.992463000	-1.709159000	-0.217684000
1	4.791293000	-2.363100000	0.463543000
1	4.834396000	-2.154467000	-1.059686000
8	5.336196000	1.522480000	0.168967000
1	5.339562000	2.214518000	-0.503423000
1	5.371553000	1.980642000	1.017360000
8	3.780221000	0.267587000	-2.223498000
1	2.875212000	0.385559000	-2.537174000
1	4.250802000	1.061138000	-2.505853000
1	4.281832000	0.549063000	2.624219000
1	2.897312000	-0.102572000	2.553327000

M06/6-311++G(d,p) optimized geometry of 1:1 Fe^{2+}−5OHFA complex in water.

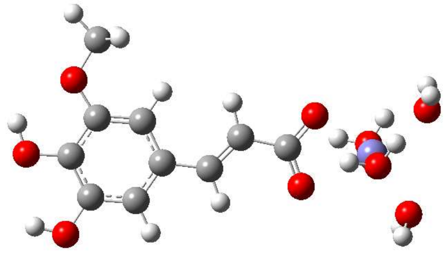

6	3.017365000	0.763212000	-0.017385000
6	4.398967000	0.809157000	-0.008431000
6	5.150364000	-0.373306000	0.004196000
6	4.503003000	-1.600286000	0.010583000
6	3.117755000	-1.652441000	0.005040000
6	2.364537000	-0.479614000	-0.010242000
6	0.915524000	-0.602068000	-0.011286000
6	0.012585000	0.385875000	-0.059753000
6	-1.434296000	0.122505000	-0.044719000
6	4.473969000	3.185477000	0.005662000
8	5.153022000	1.938717000	-0.008166000
8	-1.897344000	-1.042645000	0.064604000
8	-2.220204000	1.124809000	-0.139640000
8	6.509037000	-0.363152000	0.013936000
1	0.540420000	-1.624892000	0.033457000
1	0.301891000	1.432706000	-0.113540000
1	2.627876000	-2.622022000	0.013234000
1	2.442571000	1.682097000	-0.025333000
1	3.854306000	3.282503000	0.903437000
1	5.245210000	3.954834000	0.011662000
1	3.849970000	3.301212000	-0.886670000
1	6.823011000	0.552808000	0.015978000
26	-4.027432000	-0.002594000	0.009867000
8	-4.071345000	-0.228364000	-2.209075000
8	-5.214432000	-1.779626000	0.231325000
1	-5.020366000	-2.428971000	-0.456405000
1	-5.040950000	-2.227879000	1.068760000
8	-5.627697000	1.440469000	-0.151230000
1	-5.639980000	2.129683000	0.523897000
1	-5.669641000	1.902101000	-0.997464000
8	-4.032770000	0.207307000	2.233722000
1	-3.127433000	0.329047000	2.544891000
1	-4.505846000	0.998726000	2.517794000
1	-4.568324000	0.491368000	-2.616184000
1	-3.174007000	-0.140855000	-2.553188000
8	5.213703000	-2.763793000	0.024713000
1	6.160119000	-2.565841000	0.035639000

M06/6-311++G(d,p) optimized geometry of 1:2 Fe^{2+}−FA complex in water.

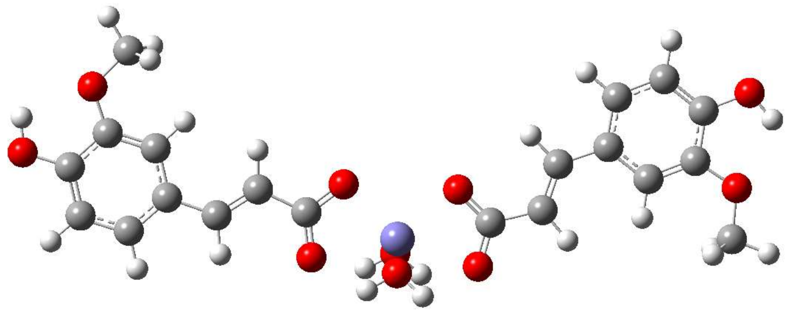

26	0.030381000	-1.479953000	0.022952000
8	-0.006295000	-2.152719000	-2.054614000
8	-0.011744000	-2.152289000	2.100472000
1	0.697210000	-2.766345000	2.325520000
1	-0.832035000	-2.594420000	2.349385000
1	-0.825407000	-2.596348000	-2.304688000
1	0.704048000	-2.765737000	-2.278134000
6	-6.606405000	1.034680000	0.010815000
6	-7.921118000	1.450801000	-0.006889000
6	-8.960702000	0.504301000	-0.065473000
6	-8.663348000	-0.843889000	-0.103977000
6	-7.339068000	-1.259964000	-0.084345000
6	-6.295976000	-0.335577000	-0.028698000
6	-4.928450000	-0.826415000	-0.014687000
6	-3.799027000	-0.105744000	0.015291000
6	-2.470497000	-0.737567000	0.020953000
6	-7.362224000	3.757207000	0.162411000
8	-8.344153000	2.741305000	0.031667000
8	-2.331214000	-1.986705000	0.016947000
8	-1.449568000	0.032542000	0.028136000
1	-4.829483000	-1.912449000	-0.033970000
1	-3.805789000	0.981412000	0.031377000
1	-7.107035000	-2.320824000	-0.112643000
1	-5.810757000	1.770392000	0.056108000
1	-6.691264000	3.770120000	-0.703377000
1	-7.903661000	4.701234000	0.214220000
1	-6.777975000	3.620561000	1.078838000
6	6.913257000	0.173180000	-0.001849000
6	8.200788000	0.667500000	-0.014681000
6	8.422832000	2.056776000	-0.043231000
6	7.350511000	2.926830000	-0.058004000
6	6.055823000	2.426575000	-0.044695000
6	5.816757000	1.052705000	-0.017153000
6	4.441201000	0.584743000	-0.006277000
6	4.009083000	-0.683868000	0.011249000

6	2.580164000	-1.028730000	0.018102000
6	9.205322000	-1.488968000	0.045389000
8	9.337520000	-0.076759000	-0.001948000
8	1.694241000	-0.115153000	0.017137000
8	2.239637000	-2.244870000	0.024448000
1	3.687577000	1.372549000	-0.014362000
1	4.695587000	-1.527123000	0.018416000
1	5.212773000	3.111625000	-0.056201000
1	6.747289000	-0.898706000	0.019589000
1	8.677291000	-1.862349000	-0.838737000
1	10.217982000	-1.890605000	0.060220000
1	8.675035000	-1.802493000	0.951231000
1	-9.478925000	-1.558963000	-0.146869000
8	-10.255657000	0.910821000	-0.083504000
1	-10.283971000	1.878310000	-0.040807000
1	7.542758000	3.994969000	-0.079186000
8	9.688751000	2.546425000	-0.055965000
1	10.313165000	1.805743000	-0.040377000

M06/6-311++G(d,p) optimized geometry of 1:2 Fe^{2+}−5OHFA complex in water.

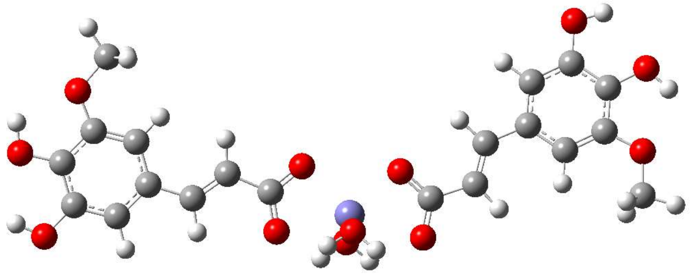

26	0.022874000	-1.618239000	0.000427000
8	-0.027574000	-2.302130000	-2.069604000
8	-0.070947000	-2.304820000	2.074380000
1	0.599250000	-2.964989000	2.287369000
1	-0.916597000	-2.700463000	2.316938000
1	-0.856955000	-2.725381000	-2.321575000
1	0.665889000	-2.942516000	-2.268957000
6	-6.463990000	1.255556000	0.166527000
6	-7.768480000	1.711718000	0.131986000
6	-8.828872000	0.821839000	-0.083911000
6	-8.568466000	-0.529377000	-0.257427000
6	-7.262106000	-0.993015000	-0.221657000
6	-6.202031000	-0.111290000	-0.014691000
6	-4.854075000	-0.657987000	-0.008210000
6	-3.699546000	0.005199000	0.138004000
6	-2.398916000	-0.681850000	0.094094000
6	-7.150908000	3.981814000	0.481271000
8	-8.160302000	3.002349000	0.289943000
8	-2.315853000	-1.931552000	-0.037722000
8	-1.343538000	0.028542000	0.183694000
1	-4.795250000	-1.737485000	-0.150829000
1	-3.660956000	1.082760000	0.277731000
1	-7.077369000	-2.053902000	-0.364418000
1	-5.650639000	1.952972000	0.329846000
1	-6.476254000	4.022940000	-0.380633000
1	-7.666142000	4.936341000	0.583789000
1	-6.574614000	3.780452000	1.390556000
6	6.955630000	-0.178383000	0.096206000
6	8.250514000	0.305508000	0.074621000
6	8.495589000	1.672676000	-0.107178000
6	7.431305000	2.549448000	-0.256490000
6	6.130186000	2.071480000	-0.227834000
6	5.880363000	0.710312000	-0.057872000
6	4.494967000	0.266073000	-0.049104000
6	4.044969000	-0.993662000	0.017709000
6	2.610511000	-1.320927000	0.010280000

6	9.229560000	-1.837228000	0.405271000
8	9.377588000	-0.437798000	0.220602000
8	1.740525000	-0.388836000	-0.005594000
8	2.255856000	-2.529280000	0.020004000
1	3.753999000	1.063192000	-0.111711000
1	4.719348000	-1.845269000	0.067377000
1	5.308879000	2.772744000	-0.345266000
1	6.773436000	-1.237487000	0.238389000
1	8.733294000	-2.297398000	-0.455770000
1	10.236969000	-2.241251000	0.501061000
1	8.663139000	-2.055661000	1.316915000
8	-10.120503000	1.241871000	-0.131490000
1	-10.147430000	2.203728000	-0.022178000
8	9.756134000	2.179507000	-0.139323000
1	10.393278000	1.458112000	-0.033187000
8	-9.582280000	-1.416848000	-0.467136000
8	7.647562000	3.884432000	-0.431215000
1	-10.428828000	-0.949810000	-0.475854000
1	8.598267000	4.061644000	-0.432595000
